# Atypically rightward cerebral asymmetry in male adults with autism stratifies individuals with and without language delay

**DOI:** 10.1002/hbm.23023

**Published:** 2015-10-23

**Authors:** Dorothea L. Floris, Meng‐Chuan Lai, Tibor Auer, Michael V. Lombardo, Christine Ecker, Bhismadev Chakrabarti, Sally J. Wheelwright, Edward T. Bullmore, Declan G.M. Murphy, Simon Baron‐Cohen, John Suckling

**Affiliations:** ^1^ Autism Research Centre Department of Psychiatry University of Cambridge Cambridge United Kingdom; ^2^ Centre for Addiction and Mental Health and Department of Psychiatry University of Toronto Toronto Canada; ^3^ Department of Psychiatry National Taiwan University Hospital and College of Medicine Taipei Taiwan; ^4^ MRC Cognition and Brain Sciences Unit Cambridge United Kingdom; ^5^ Department of Psychology and Center for Applied Neuroscience University of Cyprus Nicosia Cyprus; ^6^ Sackler Institute for Translational Neurodevelopment Department of Forensic and Neurodevelopmental Sciences Institute of Psychiatry, King's College London London United Kingdom; ^7^ Centre for Integrative Neuroscience and Neurodynamics, School of Psychology and Clinical Language Sciences, University of Reading Reading United Kingdom; ^8^ Cambridgeshire and Peterborough NHS Foundation Trust Cambridge United Kingdom; ^9^ Brain Mapping Unit Department of Psychiatry University of Cambridge Cambridge United Kingdom; ^10^ National Institute of Health Research, Cambridge Biomedical Research Centre Cambridge United Kingdom; ^11^ Behavioural and Clinical Neuroscience Institute, University of Cambridge Cambridge United Kingdom

**Keywords:** autism, cortical asymmetry, lateralization, language delay, volumetric MRI

## Abstract

In humans, both language and fine motor skills are associated with left‐hemisphere specialization, whereas visuospatial skills are associated with right‐hemisphere specialization. Individuals with autism spectrum conditions (ASC) show a profile of deficits and strengths that involves these lateralized cognitive functions. Here we test the hypothesis that regions implicated in these functions are atypically rightward lateralized in individuals with ASC and, that such atypicality is associated with functional performance. Participants included 67 male, right‐handed adults with ASC and 69 age‐ and IQ‐matched neurotypical males. We assessed group differences in structural asymmetries in cortical regions of interest with voxel‐based analysis of grey matter volumes, followed by correlational analyses with measures of language, motor and visuospatial skills. We found stronger rightward lateralization within the inferior parietal lobule and reduced leftward lateralization extending along the auditory cortex comprising the planum temporale, Heschl's gyrus, posterior supramarginal gyrus, and parietal operculum, which was more pronounced in ASC individuals with delayed language onset compared to those without. Planned correlational analyses showed that for individuals with ASC, reduced leftward asymmetry in the auditory region was associated with more childhood social reciprocity difficulties. We conclude that atypical cerebral structural asymmetry is a potential candidate neurophenotype of ASC. *Hum Brain Mapp 37:230–253, 2016*. © **2015 The Authors Human Brain Mapping Published by Wiley Periodicals, Inc.**

## INTRODUCTION

Cerebral lateralization and hemispheric specialization in structure and function are fundamental features of brain organization. At a functional level, lateralization occurs in specialized neural circuits with left‐hemisphere networks being dominant for the processing of verbal stimuli and fine motor coordination and right hemisphere systems exerting dominance for the processing of attentional, visuospatial stimuli [Gazzaniga, 1995; Gotts et al., 2013; Mesulam, 1990]. Among the most left‐lateralized language functions in typically developing individuals are syntactic [Friederici et al., 2010] and semantic processing [Binder et al., 1995; Seghier et al., 2004], word generation [Cuenod et al., 1995; Gaillard et al., 2003; Schlaggar et al., 2002] and speech production [Devlin and Watkins, 2007], speech perception [Dehaene‐Lambertz et al., 2002; Frost et al., 1999], and auditory word comprehension [Zahn et al., 2000] and phonological encoding [Coney, 2002; Shaywitz et al., 1995]. In the motor domain, the planning of complex, sequential movements [Haaland et al., 2004; Schluter et al., 1998; Verstynen et al., 2005], bimanual coordination [Jäncke et al., 2003; Serrien et al., 2003], praxis and tool use [Bohlhalter et al., 2009; Króliczak and Frey, 2009], fine motor skills (as expressed by handedness) and response selection [Weissman and Banich, 2000] are more strongly mediated by the left hemisphere. On the other hand, visuospatial abilities such as spatial reasoning (as measured by mental rotation tasks [Corballis, 1997] or the Raven's progressive matrices test [RPM; Njemanze, 2005)], spatial perception (as measured by visual search [Everts et al., 2009] or the Block Design task [Reite et al., 1993]), spatial working memory [Thomason et al., 2009] and spatial attention [Foxe et al., 2003] have mostly been attributed to right‐hemispheric processing dominance.

Anatomical substrates of these functional networks are especially evident in language‐related cortices with larger leftward volumes of perisylvian regions and auditory association areas in right‐handed males. In particular, the planum temporale (PT) has been described as the most pronounced and functionally significant asymmetry in the human brain, being 30–35% larger on the left side [Steinmetz, 1996; Steinmetz et al., 1989]. Correspondence between structure and function in other domains is less pronounced or not evident at all. Subtle leftward asymmetries have been reported in the motor cortex characterized by a deeper and more asymmetric left central sulcus (CS) [Amunts et al., 1996; Hervé et al., 2006], increased leftward neuropil in Brodmann area 4 (BA4) [Amunts et al., 1996], increased cortical thickness in the left precentral gyrus (PCG) [Luders et al., 2004] and an increased left hand motor area in right‐handers [Volkmann et al., 1998]. The inferior parietal lobule (IPL) has been suggested to be a nodal point subserving right‐lateralized attentional and spatial networks [Hugdahl and Davidson, 2003] confirmed by studies showing hemispatial neglect resulting from right‐sided IPL lesions [Kerkhoff, 2001; Na et al., 2000]. Anatomically, the IPL consists of seven cytoarchitectonically different subregions of which most parts are leftward lateralized (area “PG”), whereas a smaller posterior portion (area “PEG”) is rightward asymmetric [Eidelberg and Galaburda, 1984], and overall this region has been shown to be leftward asymmetric in males [Frederikse et al., 1999].

Lateralization in both structure and function has been explained by an evolutionary advantage ensuring more efficient transcortical integration of information and avoiding cognitive processing redundancy [Hugdahl, 2011]. In fact, leftward lateralization of functional circuits sub‐serving motor control is more beneficial for motor performance in typical children [Barber et al., 2012] and the degree of lateralization in visuospatial and language‐related networks predicts cognitive performance [Gotts et al., 2013; Mellet et al., 2014]. Furthermore, atypical, right‐ or bi‐hemispheric lateralization is more common in clinical populations with language deficits such as dyslexia [Johnson et al., 2013], stuttering [Foundas et al., 2001], specific language impairments [de Guibert et al., 2011], schizophrenia [Chance et al., 2008; Oertel‐Knöchel and Linden, 2011], and autism [Lindell and Hudry, 2013].

In particular, individuals with Autism Spectrum Conditions (ASC) show a profile of symptoms and strengths that is related to lateralized brain functions in the language, motor and visuospatial domain. For instance, even highly verbal individuals exhibit impairments in syntactic, semantic, phonological and pragmatic features of expressive and receptive language [Boucher, 2012; Kjelgaard and Tager‐Flusberg, 2001; Tager‐Flusberg and Caronna, 2007]. Alongside stereotyped behaviours, deficits in nonrepetitive motor functions arise very early on in development—including delays in motor milestones such as sitting up and starting to walk [Teitelbaum et al., 1998], clumsiness, impaired gross, and fine motor coordination [Green et al., 2009; Mostofsky et al., 2006] and problems with motor planning and planned sequencing of actions [Greenspan and Wieder, 1997]. In contrast, some individuals with ASC show intact (or even enhanced) visuospatial information processing (in terms of both perception and reasoning) as shown by superior performance on the Block Design task [Shah and Frith, 1993], Embedded Figures Task [EFT; Jolliffe and Baron‐Cohen, 1997], mental rotation tasks [Falter et al., 2008] and RPM [Dawson et al., 2007; Soulières et al., 2009]. This has been suggested by some to be underpinned by deficits in central coherence [Happé and Frith, 2006] or by hyper‐systemizing [Baron‐Cohen, 2006; Mottron et al., 2006].

This cognitive profile in individuals with ASC gave rise to an early theory [the “left hemisphere dysfunction” (LHD) theory of autism] that left‐lateralized functions are dysfunctional while right hemisphere functions remain relatively unaffected [McCann, 1981; Ricks and Wing, 1976]. Thus, prior research into hemispheric specialization in ASC has focused on identifying regions exhibiting loss or reversal of typically occurring patterns of asymmetry.

For example, studies of individuals with ASC have repeatedly reported evidence for decreased leftward, or even increased rightward, hemispheric activation during performance in expressive language tasks [Kleinhans et al., 2008; Knaus et al., 2010; Müller et al., 1999] or receptive, auditory processing [Anderson et al., 2010; Boddaert et al., 2003, 2004; Dawson et al., 1989; Müller et al., 1999]. This atypical pattern of temporal speech activation can be observed in babies [Seery et al., 2013] and toddlers [Eyler et al., 2012; Redcay and Courchesne, 2008], and it becomes more pronounced across early childhood [Eyler et al., 2012; Flagg et al., 2005]. Moreover, resting state fMRI studies confirm atypical functional rightward lateralization of numerous brain networks including language, motor, and visuospatial circuits, as well as the default mode network [Cardinale et al., 2013; Nielsen et al., 2014] and disrupted interhemispheric connectivity between language processing regions [Dinstein et al., 2011]. These atypical patterns of lateralization are functionally relevant as they are associated with poorer language abilities [Dawson et al., 1986].

Structurally, a consistent finding in verbal individuals with ASC is atypical asymmetry of the PT, with either a more symmetrical organization [Rojas et al., 2002, 2005] or rightward asymmetry [Gage et al., 2009]. In contrast, exaggerated leftward asymmetry of the PT and atypical rightward asymmetry in frontal inferior regions is especially evident in language‐impaired individuals with ASC [De Fossé et al., 2004].

In the motor domain, the most evident atypical asymmetry among people with ASC is the marked increase in the incidence of left‐and mixed‐handedness: 18–57% for left‐handedness and 17–47% for mixed‐handedness [Dane and Balci, 2007; Fein et al., 1984; Lewin et al., 1993; McManus et al., 1992; Soper et al., 1986]. Individuals who fail to establish consistent hand preference score lower on cognitive, motor, and language tasks [Hauck and Dewey, 2001]. Atypical functional lateralization on motor tasks in individuals with autism has only been investigated in few studies showing greater involvement of the right hemisphere in individuals with ASC during imitation [Dawson et al., 1983], procedural learning [D'Cruz et al., 2009] and sequence learning [Müller et al., 2004].

Atypical activation has been reported in individuals with ASC while performing visuospatial tasks. Functional studies have found decreased activation in left frontal and inferior parietal cortices alongside increased activation in bilateral superior parietal and right occipital cortex while performing the EFT [Damarla et al., 2010; Kana et al., 2013] and increased activation in right posterior parietal lobule and supramarginal gyrus (SMG) while performing mental rotation tasks [Silk et al., 2006]. However, no prior studies have examined the link between atypical (rightward) lateralization during these tasks and enhanced visuospatial performance.

Deficits in communication and language are among the core symptoms in individuals with ASC and the importance of language development is highlighted by studies showing that onset of language before the age of 2 years [Mayo et al., 2013] and the level of language at the ages 5 and 6 years [Howlin, 2003] predict functional outcome later in life in individuals with ASC. Until recently, language onset was used as a key feature to distinguish the clinical diagnoses of Asperger's syndrome versus high‐functioning autism (HFA). Although it is recognized that the broader nosological constructs autism spectrum disorders (ASD) or ASC can describe the commonality of all individuals on the spectrum, delineating heterogeneity remains one of the most critical task and challenge in autism research [Lai et al., 2013a; Lenroot and Yeung, 2013]. Finding neurobiological characteristics for subgroups in ASC is among the major aims of current research.

We have previously reported structural volumetric differences in part of the current sample between ASC individuals with and without LD [Lai et al., 2014]. However, to our knowledge no previous studies have examined lateralized differences in cortical language‐related structures between language‐delayed and non‐language‐delayed individuals with ASC. In addition, there are no previous reports of structural differences in asymmetry in regions outside the language domain in individuals with ASC. Thus, here we aim to extend the current literature on structural asymmetries in ASC by including other lateralized cognitive functions than language and differentiating between two subgroups within ASC that are defined based on one lateralized cognitive function (i.e., language). In the current study we investigated whether the functional account of the LHD theory of autism extends to structural asymmetries across grey matter (GM) in male adult individuals with ASC compared to controls using spatially restricted voxel‐based analysis. We hypothesized that atypical (i.e. rightward) patterns of asymmetry would be present in language, motor and visuospatial structures in adults with ASC, and that atypical asymmetries would be associated with worse language and motor, and better visuospatial performance. Finally, we tested the hypothesis that individuals with ASC who also had developmental language delay would show more pronounced patterns of atypical asymmetry in structures related to language function.

## MATERIALS AND METHODS

### Participants

Participants included 67 right‐handed male adults with ASC and 69 right‐handed, neurotypical male adults. Both groups did not significantly differ in age (18–43 years; ASC: mean = 26.19, SD = 6.79; controls: mean = 27.88, SD = 5.99) or full‐scale IQ (FIQ) (73–137) (see Tables 1 and 2). Participants were part of a multicentre imaging study within the UK Medical Research Council (MRC) Autism Imaging Multicentre Study (AIMS) Consortium [Ecker et al., 2012, 2013], comprising three collaborating centres: the Institute of Psychiatry, Kings College London (ASC = 38; controls = 38); the Autism Research Centre, University of Cambridge (ASC = 29; controls = 31); and the Autism Research Group, University of Oxford. In this study we focus on data acquired from Cambridge and London only as we encountered image segmentation failures due to differing signal‐to‐noise characteristics for the Oxford dataset that adversely interacted with our preferred algorithm (see below). Details of recruitment have been described elsewhere [Ecker et al., 2012, 2013; Lai et al., 2011, 2012b, 2013b; Wilson et al., 2014].

**Table 1:** Participant demographics: Individuals with ASC and neurotypicals

Characteristics	ASC (*n* = 67); mean (SD) [range]	NT (*n* = 69); mean (SD) [range]	Statistics
LD	41 no LD; 26 LD;	—;	—;
Age^a^	26.19 (6.79) [18–43]	27.88 (5.99) [18–43]	ns
Full scale IQ^a^	109.28 (14.61) [73–135]	113.93 (12.85) [77–137]	ns
Verbal IQ^a^	108.67 (14.43) [77–139]	108.77 (13.3) [71–137]	ns
Performance IQ^b^	107.6 (16.03) [73–138]	116.61 (12.32) [76–135]	NT>ASC
ADI‐R^c^	38.99 (10.0) [21–62]	—	—
Social	17.69 (5.4) [9–28]	—	—
Communication	13.78 (4.2) [8–24]	—	—
RSB	4.9 (2.23) [2–10]	—	—
ADOS^d^	11.43 (5.66) [0–24]	—	—
Communication	3.23 (1.76) [0–7]	—	—
Social	6.03 (3.19) [1–14]	—	—
RSB	1.36 (1.47) [0–6]	—	—

Abbreviations: ASC: autism spectrum condition; ADI‐R: autism diagnostic interview–revised; ADOS: autism diagnostic observation schedule; LD: language delay; NT: neurotypicals; RSB: repetitive and stereotyped behaviour.

a There were no significant differences between the ASC and control groups in age, full‐scale IQ, or verbal IQ (*P* > 0.05).

b The two groups significantly differed in performance IQ (*P* = 0.01).

c Information was available for all 67 individuals with ASC. The following cut‐off scores were used: ADI‐R Social, >10; Communication, >8; and RSB, >3.

d Information was available for 66 individuals with ASC, using a cut‐off score of 7.

**Table 2:** Participant demographics: Individuals with ASC with and without language delay

Characteristics	LD (*n* = 26); mean (SD) [range]	No‐LD (*n* = 41); mean (SD) [range]	Statistics
Age^a^	23.58 (5.37) [18–41]	27.85 (7.13) [18–43]	NT>LD
Full scale IQ^a^	106.69 (12.32) [73–128]	110.93 (15.82) [75–135]	NT>LD
Verbal IQ^b^	105.42 (11.90) [77–126]	110.73 (15.61) [79–139]	ns
Performance IQ^a^	106.15 (13.78) [73–131]	108.51 (17.41) [75–138]	NT>LD and No–LD
ADI‐R^c^	44.15 (10.04) [25–62]	35.71 (8.59) [21–55]	—
Social	20.08 (5.74) [10–28]	16.17 (4.55) [9–26]	—
Communication	15.31 (4.13) [8–24]	12.80 (4.0) [8–22]	—
RSB	4.85 (2.22) [2–10]	4.93 (2.26) [2–10]	—
ADOS^d^	12.35 (6.39) [1–24]	10.85 (5.14) [0–24]	—
Communication	3.38 (2.0) [0–7]	3.13 (1.60) [0–6]	—
Social	6.42 (3.79) [1–14]	5.78 (2.76) [1–11]	—
RSB	1.38 (1.55) [0–5]	1.35 (1.44) [0–6]	—

Abbreviations: ADI‐R: autism diagnostic interview–revised; ADOS: autism diagnostic observation schedule; LD: language delay; LD: individuals with ASC with language delay; No‐LD: individuals with ASC without language delay; NT: neurotypicals; RSB: repetitive and stereotyped behaviour.

a Individuals with LD significantly differed in performance IQ, full‐scale IQ and age form controls (*P* < 0.05).

b There were no significant differences between the ASC and control groups in verbal IQ (*P* > 0.1).

c Information was available for all 26 individuals with LD and all 41 individuals without LD. The following cut‐off scores were used: ADI‐R Social, >10; Communication, >8; and RSB, >3.

d Information was available for all 26 individuals with LD and for 40 individuals without LD, using a cut‐off score of 7.

Participants were excluded if they had: (1) a history of major psychiatric disorders, (2) severe head injury, (3) genetic disorders associated with autism (e.g., fragile X syndrome, tuberous sclerosis), (4) severe medical conditions affecting brain structure and function (e.g., epilepsy), (5) intellectual disability (IQ < 70), (6) substance‐use disorders, and (7) use of antipsychotic medications, mood stabilizers or benzodiazepines. There was no diagnosis or family history of ASC in the neurotypical (control) group. All participants gave informed written consent in accordance with the ethics approval from the National Research Ethics Committee, Suffolk, UK.

### Cognitive Measures

All individuals with ASC were clinically diagnosed with childhood autism or Asperger's syndrome according to the International Classification of Diseases‐10 [ICD‐10; World Health Organisation, 1992] criteria by a psychiatrist or clinical psychologist in the National Health Service, UK. All participants with ASC reached the diagnostic algorithm cut‐offs on the Autism Diagnostic Interview‐Revised [ADI‐R; Lord et al., 1994], but were allowed to score one point below threshold in one of the three domains (ADI‐A: Abnormalities in Reciprocal Social Interaction; ADI‐B: Abnormalities in Communication; ADI‐C: Restricted, Repetitive and Stereotyped Patterns of Behaviour). Module 4 of the Autism Diagnostic Observation Schedule [ADOS; Lord et al., 2000] was performed to assess current symptoms, but did not form part of the inclusion criteria due to potentially insufficient sensitivity among high‐functioning adult individuals. Subdomains of the ADI‐R and the ADOS were used to determine clinical symptoms in terms of deficits in social reciprocity (ADI‐R subdomain‐A; ADOS subdomain‐B), communication (ADI‐R subdomain‐B; ADOS subdomain‐A) and stereotyped, repetitive and restricted behaviour and interests (ADI‐R subdomain‐C; ADOS subdomain‐D).

The ADI‐R assessed history of language development. Language delay was defined as having onset of first words later than 24 months and/or having onset of first phrases later than 33 months. All participants were on the high‐functioning end of the spectrum (full‐scale IQ >70) as assessed by the Wechsler Abbreviated Scale of Intelligence [WASI; Wechsler, 1999]. Handedness measures were obtained using the Edinburgh Handedness Inventory [EHI; Oldfield, 1971]; only right‐handed individuals were included in the study.

Two tests for language executive functioning were administered. The FAS test [Gladsjo et al., 1999] asks individuals to generate as many words as possible starting with the letter “F” within one minute, followed by the letters “A” and “S.” The total number of words generated (excluding names, repetitions, tense changes, and plurals) is the outcome measure. The non‐word repetition task [NWR; Gathercole et al., 1994] tests individuals' phonological working memory capacity by asking the participant to repeat 28 nonwords ranging from 1 to 4 syllables that have no lexical correspondence in English. Participants are presented with standardized, prerecorded nonwords and asked to repeat them immediately. Answers are audiotaped and rated as correct if all repeated vowels, consonants and accents were the same as the stimulus. Total number of correct items constitutes the outcome measure.

Motor dexterity was assessed by the Purdue Pegboard test [PPT; Tiffin and Asher, 1948]. Participants are asked to insert small pins into holes on a board with either (a) the right hand (RH), (b) the left hand (LH), (c) with both hands alternatively (BH), or (d) to insert pins, collars, and washers using both hands alternatively (Assembly). The number of successfully placed pins within 30 s (and within 60 s for the assembly condition) is scored as the outcome measure. As focus in this study was on laterality, we additionally calculated a laterality index based on following formula: 2(RH‐LH)/(RH+LH).

Visuospatial abilities were assessed with the adult version of the EFT. We applied “Form A” consisting of 12 figures composed of a complex design and a simple shape which was part of the complex design. Participants were asked to identify the simple shape within the complex design. The time taken for the correct answer was recorded as the outcome measure. For further details on the assessment of cognitive measures see [Lai et al., 2012b; Wilson et al., 2014] (see Table 3).

**Table 3:** Overview of cognitive and behavioural measures

Symptom severity	Language	Motor	Visuospatial
**Subdomains of ADI‐R + ADOS:**			
• **Abnormal social behaviour (ADI‐A; ADOS‐B)**,	• **Non‐word repetition task**	• **Purdue pegboard test**	• **Embedded figures test**
• **Abnormal communication (ADI‐B; ADOS‐A),**	• **F‐A‐S test**		
• **Stereotyped, restricted behaviour (ADI‐C; ADOS‐D)**			

Functional measures of (a) symptom severity as measured by the ADI‐R and ADOS, (b) language as measured by the non‐word repetition task (phonological working memory), and F‐A‐S Test (verbal executive functioning), (c) motor skills as measured by the purdue pegboard test (motor dexterity), and (d) visuospatial abilities as measured by the embedded figures test.

### Structural Magnetic Resonance Imaging Acquisition

All participants underwent scanning using contemporary 3T MRI scanners fitted with an eight‐channel receive‐only radio frequency head coil (GE Medical Systems HDx, Department of Radiology, University of Cambridge; GE Medical Systems HDx, Centre for Neuroimaging Sciences, Institute of Psychiatry, Kings College London).

To guarantee standardized acquisition of structural MRI scans across centres, a validated [Ecker et al., 2012; Lai et al., 2012a, 2013b; Suckling et al., 2014] specialized acquisition protocol using quantitative imaging (DESPOT1: driven equilibrium single‐pulse estimation of T1) was applied. Spoiled gradient recalled images were acquired at two flip angles (α) from which an estimate of the absolute T_1_ value was derived at each voxel. These quantitative T_1_ maps were then used to create simulated structural T_1_‐weighted inversion recovery images, with 176 contiguous slices in 1 mm × 1 mm × 1 mm resolution, a field‐of‐view of 256 mm, a simulated repetition time/inversion time (TR/TI) of 1800/850 ms, a scaling constant ρ = 10,000 and a flip angle of 20°.

### Image Preprocessing

Voxel‐based morphometry (VBM) is the most common method for voxel‐based comparisons of GM. Here, we decided to apply a spatially restricted voxel‐wise analysis of GM asymmetry. First, this approach has the advantage of having greater sensitivity than region‐of‐interest (ROI)‐based methods in cases when not every voxel is contributing to the effect. Voxel‐wise analyses are less likely confounded by incongruent changes within ROIs and subtle differences within ROIs can be detected which might be missed when averaging across large regions of interest. Second, voxels can be regrouped flexibly and we can explore both anatomically and functionally defined ROIs whose boundaries might not correspond. Third, the majority of work with MRI on anatomical differences associated with ASC, and with differences in laterality has used VBM as the basis for their measurements. Adopting a similar methodology allows for a more natural comparison between studies. Simulated T_1_‐weighted images were preprocessed using statistical parametric mapping (SPM8; Wellcome Department of Imaging Neuroscience Group, London, UK; http://www.fil.ion.ucl.ac.uk/spm) and the VBM8 toolbox (http://dbm.neuro.uni-jena.de/vbm.html). In the following analyses, specific preprocessing steps were adopted for segmentation and normalization to meet special requirements for the analysis of asymmetry.

### Creation of Symmetrical Tissue Probability Maps

To minimize systematic left‐right biases introduced by asymmetrical tissue probability maps (TPMs), we first generated symmetric tissue priors by averaging all six tissue classes of the International Consortium for Brain Mapping (ICBM) template with their midline‐inverted (x‐flipped) counterparts. We employed a custom‐build script, which edits the data content itself without changing the header (which contains the transformation matrix). It avoids further interpolation upon writing resulted images and a consequent modification of the data.

### Creation of Symmetrical DARTEL Template

Voxel‐wise results are highly influenced by registration accuracy. Although conventional discrete cosine transform (DCT) normalization has been improved by a more flexible, high‐dimensional nonlinear diffeomorphic registration algorithm [DARTEL; Ashburner, 2007], only two previous DARTEL‐based voxel‐wise asymmetry analyses have been conducted so far [Kurth et al., 2015; Savic, 2014]. Typically, images are registered to the stereotactic coordinate system, for example that represented by the asymmetrical Montreal Neurological Institute (MNI) template provided with the SPM software, via a typically asymmetrical study‐specific DARTEL template. For asymmetry analyses, however, registration to a symmetrical template is required to ensure spatial homology between the hemispheres and prevent an artefactual increase in asymmetry due to the use of asymmetrical standard‐space templates during image registration. Here we applied an optimized preprocessing pipeline after comparing two methods (“segment then reflect” vs. “reflect then segment”; the former was deemed more optimized, see Figures S1a and S1b in Supplementary Information) to create an optimized symmetrical study‐specific template to perform high‐dimensional nonlinear registration.

### Image Segmentation Then Reflection

All original images (*N* = 136) were segmented into GM, white matter (WM), and cerebrospinal fluid (CSF) using the VBM8 toolbox. GM segmentations were then rigid‐body registered to the MNI template and reflected across the cerebral midline (*x* = 0) using the same custom‐built script as mentioned above.

### DARTEL Registration

All segmented reflected and original (non‐reflected) GM maps, rigid‐body registered to the MNI template, were then used to generate a symmetrical study‐specific template via DARTEL and were finally warped to the abovementioned symmetrical study‐specific template and then into MNI space as per standard DARTEL procedures.

A modulation step was included to retain voxel‐wise information on local volume. The final resulting images were modulated, warped, reflected (*I*_ref_) and non‐reflected (*I*_nref_) GM images in the MNI space. For the assessment of volumetric asymmetry, the laterality index (LI) was calculated at each voxel where estimates of GM volume were >0 in all images, where:

$$\text{LI}=2(I_{\text{nref}}–I_{\text{ref}})/(I_{\text{nref}}+I_{\text{ref}})$$

Positive values in the right hemisphere of the LI image indicate rightward lateralization, whereas negative values in the right side of the asymmetry image indicate leftward lateralization. Values of LI in the left hemisphere have identical magnitude, but opposite sign and were therefore excluded from further analysis. LI images were smoothed with a 4‐mm FWHM isotropic Gaussian kernel before group‐level statistical analyses.

### Regions of Interest (ROIs)

We created a set of anatomically and functionally defined ROIs using the Harvard‐Oxford atlas (fMRIB, Oxford, UK) and the online meta‐analytic database neurosynth [http://neurosynth.org; Yarkoni et al., 2011].

For deriving anatomically defined ROIs, the Harvard‐Oxford parcellation template was first coregistered (using the nearest‐neighbour method) to the symmetrical study‐specific template (in MNI space) then constrained to voxels in the study‐specific template with a tissue partial volume >0.25 to avoid edge effects between different tissue types. ROIs were selected as regions potentially subserving lateralized cognitive functions and implicated in the neurobiology of ASC, including the PT (“anatomical auditory ROI”; *k* = 1303 voxels), Broca's area (Broca; based on a conjunction of the pars opercularis and the pars triangularis, “anatomical language ROI”; *k* = 3,205 voxels), the precentral gyrus (PCG; “anatomical motor ROI”; *k* = 9,075 voxels), and inferior parietal lobule (IPL; based on a conjunction of the angular gyrus (AG) and the SMG; “anatomical visuospatial ROI”; *k* = 7,446 voxels).

For functionally defined ROIs, meta‐analytic co‐activation maps for regions functionally related to language, motor and visuospatial functions were derived from the neurosynth website (accessed June 2014) by obtaining reverse inference maps for the search terms “language” (553 studies; *k* = 8,699 voxels), “auditory” (715 studies; *k* = 7716 voxels), “motor” (1,394 studies; *k* = 16,208 voxels), and “visuospatial” (116 studies; *k* = 628 voxels). Maps were resliced to match the voxel resolution of the data, thresholded at Z ≥ 5 and binarised.

**Figure 1:**
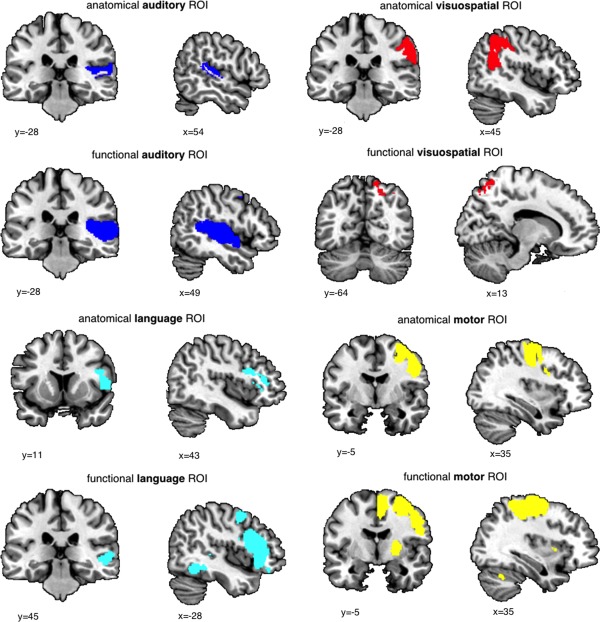
Anatomical and Functional Regions of Interest. [Color figure can be viewed in the online issue, which is available at http://wileyonlinelibrary.com.]

Each anatomical and functional ROI map was reflected along the *x* axis and the conjunction of right and left hemispheric ROIs was used for the analyses to ensure homotopic/symmetrical ROIs (see Fig. 1).

### Control ROIs

Six control ROIs (three anatomical and three functional) were additionally selected based on structures and functions that have been implicated in ASC, but not associated with atypical lateralization. Anatomical ROIs comprised: the anterior cingulate cortex (ACC) [Amaral et al., 2008; Dichter et al., 2009; Oblak et al., 2010; Thakkar et al., 2008], the caudate [Amaral et al., 2008; Langen et al., 2007; Sears et al., 1999], and the fusiform gyrus (FFG) [Amaral et al., 2008; Kwon et al., 2004; Oblak et al., 2010; van Kooten et al., 2008]. Functional ROIs were based on the neurosynth search terms “emotion regulation” (161 studies; *k* = 404 voxels) [Mazefsky et al., 2013; Samson et al., 2012], “mentalizing” (124 studies; *k* = 1,866 voxels) [Baron‐Cohen, 1995; Frith, 2001], and “sensory” (949 studies; *k* = 725 voxels) [Leekam et al., 2007; Marco et al., 2011; Tomchek and Dunn, 2007].

### Statistical Analysis

#### Group differences in total GM asymmetry

The Harvard‐Oxford atlas was coregistered to the symmetrical DARTEL template (in MNI space), thresholded at 0.25, averaged with its reflected version, binarised and split into (symmetric) right and left hemisphere ROIs. The whole right hemisphere Harvard‐Oxford ROI was used to extract values of total GM asymmetry from GM LIs. Between‐group differences were assessed by a univariate analysis of covariance (ANCOVA) including scanning centre and age as nuisance covariates.

#### Group differences in regional GM asymmetry

Between‐group differences were tested with SPM8 by regression of a general linear model at each voxel within each anatomical and each functional right hemisphere ROI separately (resulting in a total of 8 models). Group was included as a fixed factor and age and scanning centre as nuisance covariates. We also tested a separate model taking group‐by‐age interaction into account. The anatomical and functional ROIs were included as explicit masks in each model to constrain the analysis to the prespecified region. Significance levels for clusters were set at a voxel‐level cluster‐forming *P* < 0.025 and by their number of expected voxels (spatial extent threshold) according to Gaussian Random Field Theory [Chumbley and Friston, 2009]. Statistical outcomes were corrected for multiple comparisons at the cluster‐level by controlling the topological false discovery rate (FDR) at *q* < 0.05. Anatomical subregions within significant clusters were labelled where it overlapped with specific regions of the Harvard‐Oxford atlas. Post‐hoc one‐sample *t* tests were conducted in SPSS to explain the within‐group direction of effect in the cluster.

Subsequently, it was determined whether significant group differences in laterality were driven by increased rightward or decreased leftward volume. For this, the modulated, warped, non‐reflected (*I*_nref_) GM images in MNI space were smoothed with a 4‐mm FWHM kernel. Significant clusters were then binarised and reflected resulting in homotopic right‐ and left‐hemisphere ROIs. Mean values in the GM voxels were extracted from each ROI, multiplied with total ROI volume and compared using an ANCOVA (with age, scanner, and total GM volume [derived from native‐space partial volume estimates] as covariates) between the two groups in SPSS. The same procedure was applied with the control ROIs.

#### Correlation between atypical GM asymmetry and behavioural/cognitive measures

Examination of the relationship between atypically lateralized regions and historical and current symptoms of ASC was conducted for each significant cluster. One‐tailed partial correlations were calculated for the atypically lateralized region with functional measures in the ASC group, controlling for the effects of age and centre: a) the anatomical (PT and Broca) and functional (“language” and “auditory”) language ROI LI and the social and communication sub‐scores of the ADI‐R and ADOS, the FAS and the NWR; b) the anatomical (PCG) and functional (“motor”) motor ROI LI and the repetitive behaviour sub‐scores of the ADI‐R and the PPT; c) the anatomical (IPL) and functional (“visuospatial”) visuospatial ROI LI and the EFT. Significance threshold was corrected for multiple comparisons only within the three groups (i.e., between anatomical and functional ROI pairs) resulting in a *P* = 0.025, but not between them, as the correlation analyses were testing different effects. Correlations with the ADOS, FAS, NWR, PPT, and EFT were conducted in a slightly smaller sample due to missing data (ADOS: 1 ASC; FAS: 1 control, 1 ASC; NWR: 3 controls, 3 ASC; PPT: 1 control, 1 ASC; EFT: 2 controls, 3 ASC) and the exclusion of one outlier in the ASC group with extreme scores on the FAS. Analyses were done with SPSS (version 21, SPSS).

#### Language‐delayed vs. non‐language‐delayed individuals with ASC

Any significant result involving language or auditory ROIs was followed up by testing the same model with diagnosis/group (controls vs. language‐delayed vs. nonlanguage‐delayed) as a fixed factor.

To test the effect of language delay (LD), we conducted MANCOVAs including cognitive language measures (as specified above) as dependent variables. Main effects of diagnosis (LD vs. no LD) and auditory/language LIs along with the diagnosis‐by‐LI interaction were investigated covarying for age and scanner. Any significant result was followed up with within‐group correlational analyses.

## RESULTS

### Participant Characteristics and Task Performance

ASC and control groups did not significantly differ in their age (*t*(134) = 1.541, *P* = 0.126), verbal IQ (VIQ) (*t*(134) = −0.041, *P* = 0.968) and handedness (*U* = 2,089.5, *z* = −0.761, *P* = 0.447). Difference in full‐scale IQ (FIQ) (*t*(134) = 1.97, *P* = 0.051), however, trended toward significance. There was a significant group difference in performance IQ (PIQ) (*t*(123.85) = 3.669, *P* < 0.001). Individuals with ASC scored significantly lower than controls on the NWR (*F*(1,127) = 5.709, *P* = 0.018) and on three subtests of the PPT (RH: *F*(1,131) = 9.765, *P* = 0.002; LH: *F*(1,131) = 6.315, *P* = 0.013; BH: *F*(1,131) = 1.026, *P* = 0.313; Assembly: *F*(1,131) = 19.97, *P* < 0.001). There were no group differences in laterality on the PPT (*F*(1,131) = 0.023, *P* = 0.881). There were no group differences in performance on the FAS (*F*(1,130) = 1.886, *P* = 0.172) and in reaction time on the EFT (*F*(1,128) = 0.68, *P* = 0.411).

### Group Differences in Total GM Asymmetry

Individuals with ASC and controls did not differ in hemispheric LI, calculated from total grey matter (*F*(1,131) = 0.608, *P* = 0.437). There was no significant group‐by‐age interaction (*F*(1,131) = 0.002, *P* = 0.961).

### Group Differences in Regional GM Asymmetry

#### Anatomical ROIs

**Figure 2:**
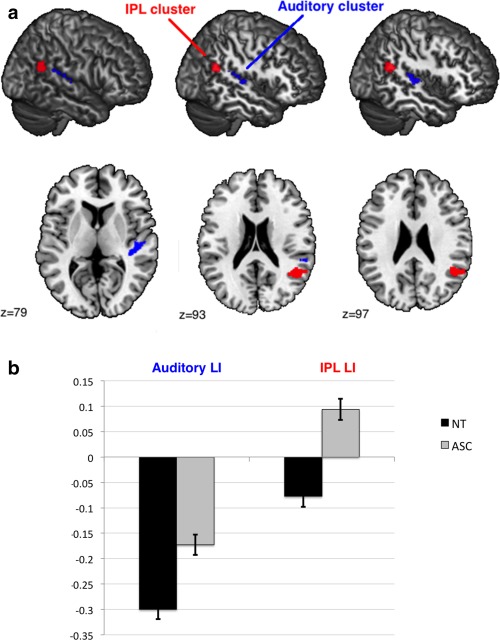
(a) Significant cluster (ASC vs. controls). Sagittal slices of the MNI stereotactic atlas with superimposed significant between‐group differences in LI covering perisylvian regions. Red: IPL cluster; Blue: Auditory cluster. (**b**) Means and standard deviations of laterality indices extracted from the auditory cluster and the IPL cluster. Positive values indicate rightward asymmetry, and negative values indicate leftward asymmetry (with arbitrary unit). Controls (NT) show leftward asymmetry in both clusters, whereas individuals with ASC show reduced leftward asymmetry in the auditory cluster and reversed rightward asymmetry in the IPL cluster. [Color figure can be viewed in the online issue, which is available at http://wileyonlinelibrary.com.]

**Figure 3:**
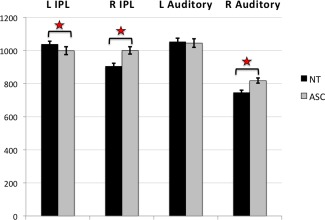
Group differences in left and right IPL and Auditory cluster volume. Means and SD of volume (in mm^3^) extracted from left and right auditory and IPL clusters. Differences in the IPL cluster were driven by larger rightward and smaller leftward volumes, whereas only by larger rightward volume in the auditory cluster in individuals with ASC. [Color figure can be viewed in the online issue, which is available at http://wileyonlinelibrary.com.]

**Figure 4:**
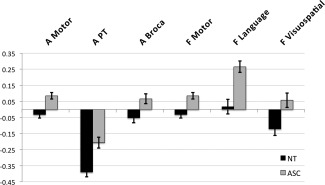
Group differences in peak voxels for non‐significant ROIs. Abbreviations: A Motor: anatomical motor ROI; A PT: anatomical auditory ROI (planum temporale); A Broca: anatomical language ROI; F Motor: functional motor ROI; F Language: functional language ROI; F Visuospatial: functional visuospatial ROI.

Between‐group voxel‐wise analysis of LI in anatomical ROIs revealed one significant cluster within the IPL ROI (cluster size *k*_e_ = 431 voxels, cluster‐level FDR‐corrected *q* = 0.01, peak‐voxel MNI coordinate [45, −54, 22], *T* = 4.61) with individuals with ASC showing increased rightward asymmetry compared to controls (see Fig. 2a). The cluster made up 5.79% of the IPL ROI. Post‐hoc one‐sample *t* tests revealed that individuals with ASC had rightward asymmetry in the IPL cluster (*t*(66) = 4.480, *P* < 0.001), whereas controls exhibited leftward asymmetry (*t*(68) = −3.684, *P* < 0.001) (see Fig. 2b). This result was driven by both rightward increases and leftward reductions in IPL volume (group difference left IPL: *F*(1,131) = 6.092, *P* = 0.015; group difference right IPL: *F*(1,131) = 13.866, *P* < 0.001) (see Fig. 3). There was no cluster showing a significant group‐by‐age interaction (cluster‐level FDR‐corrected *q* = 0.894). This result remained significant when including full‐scale IQ as an additional covariate (cluster size *k_e_* = 474 voxels, cluster‐level FDR‐corrected *q* = 0.009, peak‐voxel MNI coordinate [51, −49, 23], *T* = 5). There were no significant differences for the PT (cluster size *k_e_* = 141 voxels, cluster‐level FDR‐corrected *q* = 0.405, peak‐voxel MNI coordinate [45, −26, 7], *T* = 4.19), Broca (cluster size *k_e_* = 11 voxels, cluster‐level FDR‐corrected *q* = 0.902, peak‐voxel MNI coordinate [55, 38, 5], *T* = 2.85) and PCG (cluster size *k_e_* = 180 voxels, cluster‐level FDR‐corrected *q* = 0.572, peak‐voxel MNI coordinate [55, 3, 37], *T* = 3.86) ROIs (see Fig. 4).

#### Functional ROIs

Between‐group voxel‐wise analysis of LI in functional ROIs revealed one significant cluster within the auditory ROI (cluster size *k_e_* = 446 voxels, cluster‐level FDR‐corrected *q* = 0.006, peak‐voxel MNI coordinate [45, −26, 7], *T* = 4.19). Individuals with ASC showed decreased leftward asymmetry compared to controls in a cluster within the functional auditory ROI (see Fig. 2a). The cluster made up 5.78% of the auditory ROI. Based on the Harvard‐Oxford atlas the cluster involved parts of the posterior supramarginal gyrus (pSMG), parietal operculum (PO), PT and Heschl's gyrus (HG). Post‐hoc one‐sample *t* tests revealed that both individuals with ASC (*t*(66) = −8.577, *P* < 0.001) and controls (*t*(68) = −15.608, *P* < 0.001) had leftward asymmetry in the auditory cluster (see Fig. 2b). This result was driven by rightward increases in auditory cortex volume (group difference left auditory cortex: *F*(1,131) = 1.317, *P* = 0.253; group difference right auditory cortex: *F*(1,131) = 11.563, *P* = 0.001) (see Fig. 3). There was no cluster showing a significant group‐by‐age interaction (cluster‐level FDR‐corrected *q* = 0.895). This result remained significant when including full‐scale IQ as an additional covariate (cluster size *k_e_* = 434 voxels, cluster‐level FDR‐corrected *q* = 0.01, peak‐voxel MNI coordinate [45, −26, 7], *T* = 4.1). There were no significant differences for the language (cluster size *k_e_* = 20 voxels, cluster‐level FDR‐corrected *q* = 0.897, peak‐voxel MNI coordinate [51, −49, 22], *T* = 4.17), motor (cluster size *k_e_* = 169 voxels, cluster‐level FDR‐corrected *q* = 0.486, peak‐voxel MNI coordinate [55, 3, 37], *T* = 3.86) and visuospatial (cluster size *k_e_* = 35 voxels, cluster‐level FDR‐corrected *q* = 0.878, peak‐voxel MNI coordinate [25, −66, 45], *T* = 2.86) ROIs (see Fig. 4).

For non‐thresholded, descriptive *t*‐maps, see Figures a and b.

#### Control ROIs

For anatomical control ROIs there were no significant group differences for the ACC (cluster size *k*_e_ = 19 voxels, cluster‐level FDR‐corrected *q* = 0.476, peak‐voxel MNI coordinate [3, 38, −10], *T* = 3.09), the caudate (cluster size *k*_e_ = 11 voxels, cluster‐level FDR‐corrected *q* = 0.901, peak‐voxel MNI coordinate [18, 19, 13], *T* = 2.78) or FFG (cluster size *k*_e_ = 7 voxels, cluster‐level FDR‐corrected *q* = 0.902, peak‐voxel MNI coordinate [45, −57, −16], *T* = 2.77) ROIs.

For the functional control ROIs there were no significant group differences either for the “emotion regulation” (no supra‐threshold clusters), “mentalizing” (cluster size *k*_e_ = 4 voxels, cluster‐level FDR‐corrected *q* = 0.761, peak‐voxel MNI coordinate [43, −52, 15], *T* = 2.48) or “sensory” (cluster size *k*_e_ = 127 voxels, cluster‐level FDR‐corrected *q* = 0.119, peak‐voxel MNI coordinate [48, −23, 7], *T* = 3.81) ROIs.

### Correlations With Cognitive Measures in Individuals With ASC

**Figure 5:**
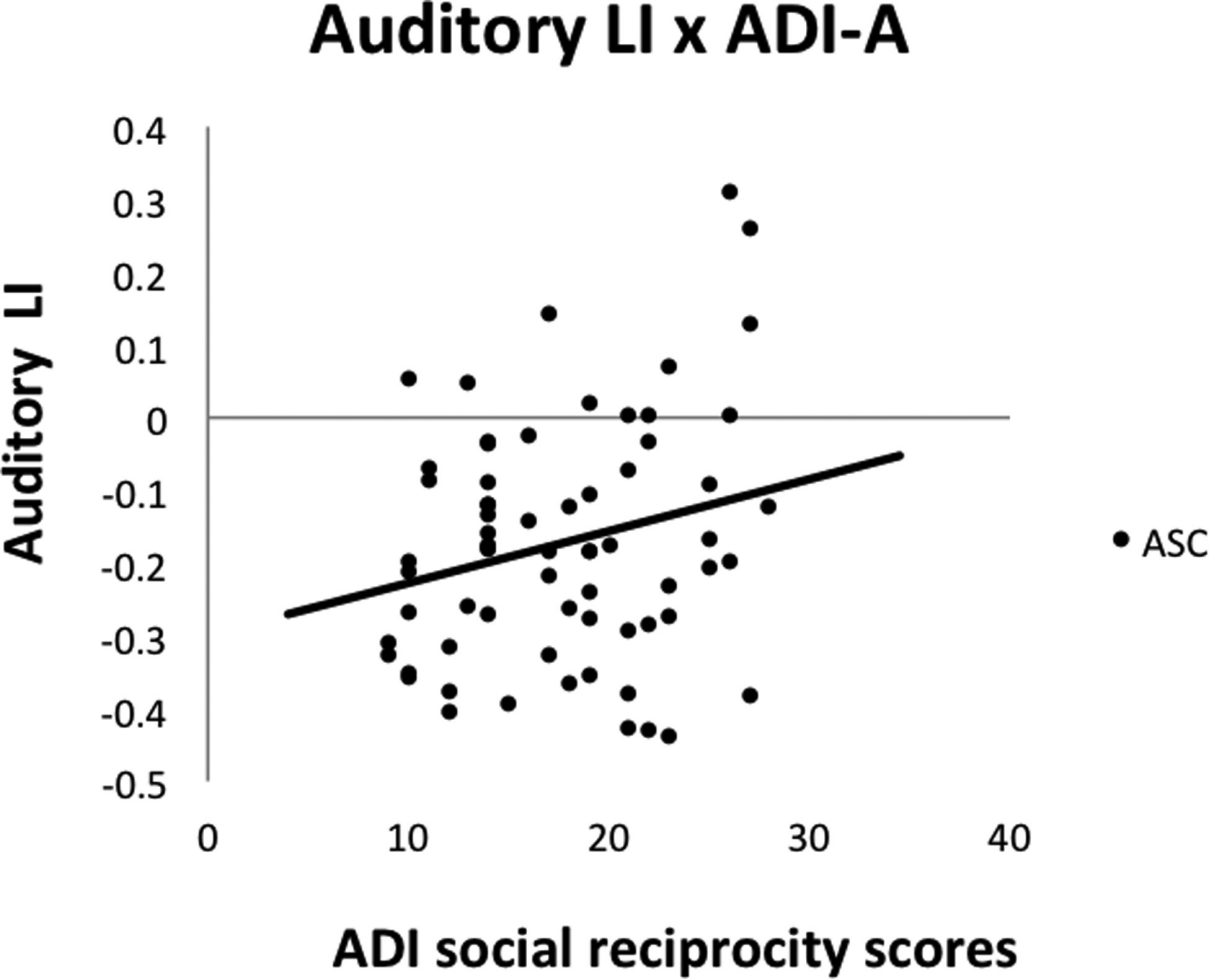
Relationship between Auditory LI and symptom severity. Positive values indicate rightward asymmetry, and negative values indicate leftward asymmetry (arbitrary unit). There was a positive correlation between abnormal social reciprocity scores on the ADI‐R and asymmetry of the auditory cluster, indicating more childhood symptoms with reduced leftward / stronger rightward asymmetry.

Given the PT's functional significance and implication in ASC, we calculated the overlap between the auditory cluster and the PT by finding the conjunction between the cluster and the PT sub‐region derived from the Harvard‐Oxford parcellation template (PT_Auditory_ LI). We then correlated the LI in the whole auditory ROI, and the PT_Auditory_ LI, with functional measures related to social communication. The LI in the whole auditory cluster was positively correlated with the ADI‐R subscore for abnormal social reciprocity (Pearson's *r* = 0.253, *P* = 0.021; controlling for FIQ: *r* = 0.251, *P* = 0.023), that reduced leftward asymmetry was associated with more childhood social symptoms in individuals with ASC (see Fig. 5). The same correlation was also significant for the PT_Auditory_ LI (*r* = 0.249, *P* = 0.023; controlling for FIQ: *r* = 0.260, *P* = 0.019). The remaining targeted comparisons were not significant (see Table 4).

**Table 4:** Associations between significant clusters and functional measures

	Auditory LI	PT_Auditory_ LI	IPL LI
ADI‐A	*r* = 0.253, *P* = 0.021*	*r* = 0.249, *P* = 0.023*	—
ADI‐B	*r* = 0.118, *P* = 0.174	*r* = 0.145, *P* = 0.125	—
ADOS‐A	*r* = 0.165, *P* = 0.096	*r* = 0.200, *P* = 0.057	—
ADOS‐B	*r* = 0.130, *P* = 0.153	*r* = 0.143, *P* = 0.129	—
NWR	*r* = −0.059, *P* = 0.325	*r* = −0.002, *P* = 0.494	—
FAS	*r* = −0.098, *P* = 0.222	*r* = −0.065, *P* = 0.306	—
EFT	—	—	*r* = 0.038, *P* = 0.385

Correlation between the Auditory LI and its conjunction with the PT (PT_Auditory_ LI) with measures of social communication (ADI‐A, ADI‐B, ADOS‐A, ADOS‐B, NWR, FAS) and the IPL LI with a visuospatial task (EFT).

### Language‐delayed vs. Non‐language‐delayed Individuals With ASC

#### Participant characteristics and task performance

Groups significantly differed in age from each other (*F*(2,133) = 4.972, *P* = 0.008), with individuals with ASC with LD being younger than controls (ASC‐LD: Mean = 23.58, SD = 5.37; controls: Mean = 27.88, SD = 5.99; *t*(93) = −3.211, *P* = 0.002), but there was no difference between individuals with ASC without LD and controls (ASC‐No‐LD: Mean = 27.85, SD = 7.13; *t*(108) = −0.024, *P* = 0.981). ASC individuals with and without LD and controls did not significantly differ in VIQ from each other (*F*(2,136) = −1.178, *P* = 0.311). There was a trending difference in FIQ (*F*(2,133) = 2.704, *P* = 0.071), driven by a significant difference between individuals with ASC with LD and controls (*t*(93) = −2.473, *P* = 0.015), but there was no difference between individuals with ASC without LD and controls (*t*(108) = −1.085, *P* = 0.280). There was a significant difference in PIQ (*F*(2,133) = 6.969, *P* = 0.001) between the three groups, which was present both between controls and individuals with ASC without LD (*t*(63.99) = −2.614, *P* = 0.011) and controls and individuals with ASC with LD (*t*(93) = −3.570, *P* = 0.001). There was no difference in handedness (*χ* ^2^(2) = 2.681, *P* = 0.262) between the three groups. Based on these differences in demographic data, analyses were repeated in an age‐ and IQ‐matched sub‐sample (see Supporting Information).

Individuals with ASC with and without LD scored significantly lower than controls on the NWR (*F*(2,126) = 3.664, *P* = 0.028). This difference was driven by individuals with ASC with LD (*F*(1,87) = 7.641, *P* = 0.007) and there was no difference between individuals with ASC without LD and controls (*F*(1,103) = 2.481, *P* = 0.118). There was also a significant group differences in FAS performance between the three groups (*F*(2,130) = 5.389, *P* = 0.006). This was driven by individuals with ASC with LD (*t*(91) = −3.154, *P* = 0.002), but there was no significant difference between individuals without LD and controls (*t*(106) = −0.002, *P* = 0.999).

#### Group differences in “auditory” GM asymmetry

Comparing controls with individuals with ASC with and without early language delay using a polynomial contrast resulted in a significant linear trend (*P* < 0.001) across the three groups, with individuals with ASC without language delay showing an intermediate position between neurotypicals and ASC individuals with language delay.

**Figure 6:**
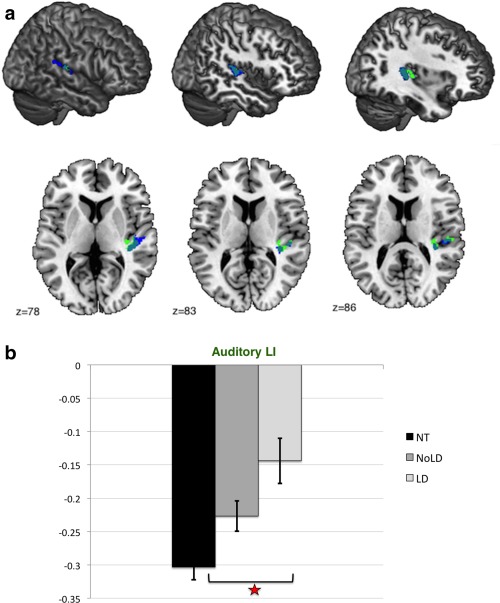
(a) Significant cluster (ASC with LD vs. controls). Sagittal slices of the MNI stereotactic atlas with superimposed significant between‐group differences in the auditory LI showing the overlap between significant clusters. Blue: Auditory cluster (all ASC‐controls); Green: Auditory cluster (ASC with LD‐controls). (**b**) Group differences in Auditory LI between individuals with ASC with and without LD and controls. Abbreviations: NT: neurotypicals (controls); NoLD: individuals with ASC without language delay; LD: individuals with ASC with language delay means and standard deviations of laterality indices extracted from the auditory cluster (ASC with LD vs. controls). Positive values indicate rightward asymmetry, and negative values indicate leftward asymmetry (with arbitrary unit). Individuals without language delay (NoLD) show an intermediate position between individuals with language delay (LD) and controls (NT) for the values extracted from the auditory cluster. [Color figure can be viewed in the online issue, which is available at http://wileyonlinelibrary.com.]

On the basis of this significant trend result, we tested the model including the auditory ROI with diagnosis as a fixed factor. Between‐group voxel‐wise analysis of LI in the functional auditory ROI revealed one significant cluster (cluster size *k*_e_ = 444 voxels, cluster‐level FDR‐corrected *q* = 0.015, peak‐voxel MNI coordinate [37, −31, 10], *T* = 3.48) between individuals with ASC with LD compared to controls (see Fig. 6a). The cluster made up 5.75% of the auditory ROI. This result was driven by rightward increases in auditory cortex volume (group difference left auditory cortex: *F*(1,90) = 2.656, *P* = 0.107; group difference right auditory cortex: *F*(1,90) = 6.068, *P* = 0.016). This result remained significant in an age‐ and IQ‐matched sub‐sample (see Supporting Information). There was no significant difference between individuals with ASC without LD and controls, or between the two subgroups with ASC. A polynomial contrast showed that individuals with ASC without LD had a significant intermediate position (*P* < 0.001) (see Fig. 6b).

#### Associations with cognitive measures in individuals with ASC with and without LD

**Figure 7:**
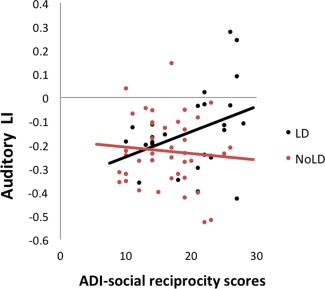
Differential association between the Auditory LI and social symptoms in individuals with ASC with and without LD. Positive values indicate rightward asymmetry, and negative values indicate leftward asymmetry (arbitrary unit). There was a positive correlation between abnormal social reciprocity scores on the ADI‐R and asymmetry of the auditory cluster, indicating greater childhood symptoms with reduced leftward / stronger rightward asymmetry in ASC individuals with LD, but not in those without LD. [Color figure can be viewed in the online issue, which is available at http://wileyonlinelibrary.com.]

There was a trending diagnosis‐by‐LI interaction for the social reciprocity subdomain of the ADI (*F*(1,61) = 1.590, *P* = 0.066). Follow‐up within‐diagnosis correlational analysis showed that this association between the auditory LI and the ADI scores was significant within individuals with ASC with LD (*r* = 0.373, *P* = 0.036), but not in individuals with ASC without LD (*r* = −0.092, *P* = 0.289) (see Fig. 7). There were no significant diagnosis‐by‐LI interactions for any other language measures.

**Figure 8a:**
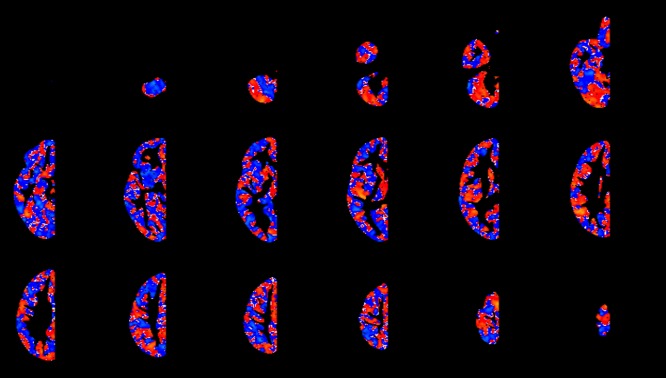
Non‐thresholded, descriptive t‐maps. Unthresholded t‐map of the ASC‐Control contrasts. Regions where ASC has larger LI values are shown in red, whereas regions where controls have larger LI values than ASC are shown in blue. [Color figure can be viewed in the online issue, which is available at http://wileyonlinelibrary.com.]

**Figure 8b:**
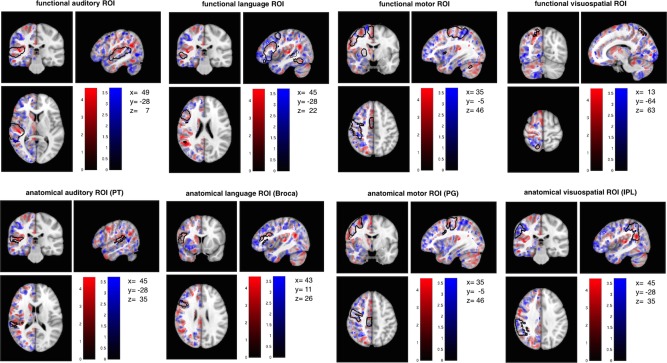
Unthresholded t‐maps within each anatomical and structural ROI. Unthresholded t‐map of the ASC‐Control contrasts. Regions where ASC has larger LI values are shown in red, whereas regions where controls have larger LI values than ASC are shown in blue. [Color figure can be viewed in the online issue, which is available at http://wileyonlinelibrary.com.]

## DISCUSSION

Our objective was to identify whether male adult individuals with ASC have atypical structural lateralization, and if there are associations between atypical asymmetry and language, motor, and visuospatial functions. Comparing individuals with ASC to a matched neurotypical control group, we found significant reductions and reversals of typically leftward asymmetry extending along the sylvian fissure in the auditory cortex and inferior parietal lobule. Also, within the auditory cluster there was further evidence for an association with autistic characteristics, and specifically with childhood social reciprocity.

### Atypical Asymmetry in Auditory Regions

The HG, PT, and PO constitute the core of the temporal speech regions with well‐established leftward asymmetries in typically developing individuals. Our results are in line with findings showing reductions of structural asymmetry [Rojas et al., 2002, 2005] and decreased leftward activation [Anderson et al., 2010; Eyler et al., 2012; Flagg et al., 2005; Gage et al., 2009; Kleinhans et al., 2008; Knaus et al., 2010; Lindell and Hudry, 2013] in posterior language regions in individuals with ASC. Among the key components of the language system, auditory processing plays a particularly crucial role in the acquisition of language in infant development. The ability to detect, distinguish, and categorize speech sounds is the prerequisite for building accurate speech representations and eventually producing meaningful speech [Dockrell and Messer, 1999]. A range of studies shows that early auditory processing has predictive value for later language outcomes [Lombardo et al., 2015; Molfese and Molfese, 1997; Trehub and Henderson, 1996]. Atypical lateralization in auditory association areas in ASC has been attributed to dysfunction of temporal regions specialized in word perception, long‐term representations and integration of complex sounds, which eventually results in deficits in language comprehension and production [Boddaert et al., 2004]. Here we show that atypical lateralization of temporal, auditory regions are also represented on a structural level.

In contrast to our findings, two studies have reported increased leftward asymmetry [De Fossé et al., 2004; Herbert et al., 2002] in the PT of individuals with ASC. It is still to be established whether this is specific to a particular subgroup of individuals with ASC, especially in the light of the finding that in neurotypical individuals exaggerated leftward asymmetries of the PT have been associated with enhanced abilities in the processing of auditory stimuli such as perfect pitch [Schlaug et al., 1995] rather than with deficits.

Inconsistency in PT asymmetry results in ASC might reflect differences in sample characteristics such as age, handedness, and particularly degree of language impairment, as well as methodological variation [Lenroot and Yeung, 2013]. It is likely that different degrees of atypical asymmetry in distinct regions explain part of the variability in language ability in individuals with ASC and adds to the idea that heterogeneity in the clinical characteristics of ASC may reflect differential neurodevelopmental pathways and brain maturational processes. Subgrouping may thus be an essential part of any understanding of ASC [Lai et al., 2013a].

As functional neuroimaging studies suggest an important role of the PT in language processing [Tzourio et al., 1998; Wise et al., 2001], it is surprising that we did not find an association between atypical lateralization in the auditory cluster with any measure of language functioning. Instead, we found that rightward asymmetry of regions related to the core auditory processing areas, including the PT, were correlated with more childhood social reciprocity symptoms. In line with this, Coffey‐Corina et al. [2008] reported that toddlers with ASC with fewer social symptoms exhibited similar left‐lateralized event‐related potential (ERP) responses to auditory stimuli to typical toddlers, whereas toddlers with ASC with more severe social symptoms showed rightward lateralization. Also, there is a very close interrelation between social and communicative dimensions, which has recently been acknowledged in DSM‐5 by their conflation into one symptom domain. Social behaviour relies on communication and communication is inherently social. This close functional relationship might also be subserved by the same neuronal networks and explain why alterations of asymmetry in perisylvian regions are associated with childhood social deficits in individuals with ASC. In fact, social and communication symptoms were highly correlated with each other on both the ADOS (*r* = 0.730, *P* < 0.001) and the ADI‐R (*r* = 0.585, *P* < 0.001). However, it is interesting that only childhood symptoms showed an association with atypical asymmetry raising the possibility that symptoms were more pronounced at ages 4–5 years and individuals might have improved in their social‐communication skills, even though the underlying neurobiological alterations remain present.

Current measures of language functioning did not show any significant association with atypical asymmetry, which may owe to a lack of specificity of our measures. NWR and FAS measure phonological working memory and verbal executive function which are functions subserved by frontal and parietal networks [Andreasen et al., 1995; Paulesu et al., 1993; Petrides et al., 1993] beyond the auditory cortex, which might be more sensitive to measures of language processing and comprehension.

We did not observe atypical rightward asymmetry in inferior frontal and other language‐specific regions in individuals with ASC. Broca's area has repeatedly been shown to be right‐lateralized in language impaired individuals with ASC [De Fossé et al., 2004; Herbert et al., 2002]. Our sample did not include individuals with clinically significant language impairments. It is likely that this alteration in inferior frontal asymmetry is specific to the ASC subgroup with clinically significant language impairments.

### Atypical Asymmetry in the Inferior Parietal Lobule (IPL)

We found reversed rightward asymmetry in the IPL in individuals with ASC compared to controls, however without behavioural correlation with a visuospatial task. Previous studies are in line with this result showing that typically developing males usually exhibit stronger leftward asymmetry in the inferior parietal lobe [Frederikse et al., 1999], which has been linked to visuospatial superiority in males. Still, spatial processing is not based on one focal region, but rather is the result of a complex interplay of differentially lateralized regions making up a right‐lateralized network of which some components are potentially atypically lateralized in autism. Of these, the IPL has been considered a nodal point of an attention network underlying spatial processing [Heilman and Van Den Abell, 1980]. Both controls and individuals with ASC activate right superior and inferior parietal lobule and supramarginal gyrus during visuospatial processing [Silk et al., 2006]. Differences have been observed in that controls recruit interconnected frontal‐parietal‐occipital networks for visuospatial processing [Gotts et al., 2013; Kana et al., 2013], whereas individuals with ASC rely more on posterior parietal structures rather than integrating perceptual and executive processes when it comes to performing spatial tasks. Atypical integration of specialized regions and reversal of direction of specialization might underlie differences in perceptual functioning. It is still to be determined whether this reversed pattern of asymmetry explains intact or even enhanced visuospatial processing in individuals with ASC, or whether it is related to deficits in other cognitive domains. It will be informative to further examine lateralization of structures involved in visuospatial and attentional processes in the subgroup of individuals with ASC who exhibit superior visuospatial abilities.

Inferring the engagement of specific cognitive processes based on the activation in (or here structural alterations in) specific brain regions [“reverse inference”; Poldrack, 2006] is one caveat of defining ROIs anatomically, making it difficult to establish associated function with the observed atypicality. Here we focussed on the IPLs role in spatial processing, however it is important to also emphasize its involvement in a range of other functions that are atypical in ASC such as imagery and imitation of actions [Stephan et al., 1995; Williams et al., 2006], gaze processing [Calder et al., 2007; Wicker et al., 1998], semantic processing [Binder and Desai, 2011; Démonet et al., 1992; Wang et al., 2010], and mathematical functions and number processing [Critchley, 1966; Dehaene et al., 2003; Ischebeck et al., 2009]. In particular, the temporoparietal junction (TPJ) has been implicated in social cognition such as empathy, social attention and theory of mind [Buckner et al., 2008; Decety and Lamm, 2007; Mar, 2011; Nummenmaa and Calder, 2009], which constitute core symptoms of ASC. Thus, being one of the most highly connected hubs in the brain [Tomasi and Volkow, 2011], atypical asymmetry in the IPL is likely to contribute to multiple domains of impairment in ASC. Strikingly, reversal of typical leftward asymmetry of the AG has also been reported in schizophrenia [Niznikiewicz et al., 2000], another neurodevelopmental condition involving atypical lateralization. The common occurrence of these atypical asymmetries in conditions with overlapping symptoms confirms that underlying neural aberrations are systematic and not merely random changes [Herbert et al., 2005]. Leftward asymmetry in the AG and PT was shown to be correlated in typically developing adults [Eidelberg and Galaburda, 1984] lending support to the notion that they form part of a common functional network. Future studies with clear hypotheses about atypical asymmetry in the IPL in ASC should include specific cognitive tasks that are associated with activation in this region.

### Lateralization of Motor Functions

We did not find atypical rightward asymmetries in regions integral to motor functioning. Structural asymmetries in the motor cortex are very subtle in general and are most marked in the hand motor region [Volkmann et al., 1998]. Functional specialization of typical sensorimotor networks is left lateralized and correlates with activity in the same regions during a hand movement task [De Luca et al., 2005]. One study has so far provided evidence for rightward alterations of asymmetry in widespread functional circuits (also comprising the sensorimotor resting network) in individuals with ASC [Cardinale et al., 2013]. It is still to be established whether alterations in functional sensorimotor specialization underlie repetitive and nonrepetitive motor deficits in ASC such as clumsiness, fine‐motor skills, balance, and gait [Fournier et al., 2010; Gowen and Hamilton, 2013].

In the context of motor asymmetries, another promising area to be investigated is whether left‐handed individuals with ASC show even more pronounced asymmetry in these regions, in association with poorer cognitive and behavioural performance. Previously, we found that left‐handed, but not right‐handed, adolescents with ASC differed from neurotypical adolescents in asymmetry in the central region of the corpus callosum which projects to the sensorimotor and posterior parietal cortex, indicating that atypical asymmetries might be even more pronounced in left‐handers [Floris et al., 2013].

### Language Delay

Results showed that individuals with ASC with early LD exhibit stronger deviations from typically leftward asymmetry in cortical auditory regions than those without LD. It has previously been shown that different developmental language profiles are associated with brain morphological alterations in ASC, such as structural differences between male adult individuals with and without LD [Lai et al., 2014] or early brain overgrowth in males with regressive autism [Nordahl et al., 2011] [which might affect typical patterns of lateralization; Rilling and Insel, 1999; Ringo, 1991]. Lai et al. [2014] found reduced volume in individuals with ASC with a history of LD compared to those without LD in regions including the superior temporal gyrus (STG), middle temporal gyrus (MTG), superior temporal sulcus (STS), and temporal pole. These regions overlap with present regions showing lateralized differences and it is possible that these noted volumetric differences are driven by underlying lateralized differences between the two subgroups of ASC. Also, Lombardo et al. [2015] showed that hypoactivation in superior temporal cortices during passive speech perception can potentially serve as a neurophenotype specifying individuals with ASC with poor developmental outcome.

In another study, comparing laterality differences in language‐based subgroups within ASC, Rinehart et al. [2002] compared the laterality of executive function task performance in individuals with ASC and found left‐lateralized deficits in the language‐delayed subgroup only; the authors proposed that this may be associated with the timing at which a shift in lateralization occurs in relation to speech onset. Also, Escalante‐Mead et al. [2003] reported reduced rates of established hand preference in individuals with autism who had early language delay. No previous studies have however compared subgroups within ASC in terms of structural lateralization. Here we show that the degree of atypical lateralization constitutes a candidate biomarker of different subgroups with different language profiles in ASC. Future studies of lateralization in ASC should thus differentiate between different developmental pathways, for example taking language delay or regression into consideration.

There was also a trending interaction between diagnosis and cortical auditory asymmetry in relation to abnormal social functioning. Increased rightward asymmetry seems to be associated with more atypical social behaviour in language‐delayed individuals only. This is interesting in the light of reports that social deficits are more pronounced and social motivation is decreased in individuals with ASC with LD [Macintosh and Dissanayake, 2004; Szatmari et al., 1995; Verté et al., 2006]. Also, Lai et al. [2014] argue that LD constitutes a marker of impaired social development. Atypical asymmetry in perisylvian cortical areas potentially mediates the observed effects of LD on social‐communicative development.

Observed differences between individuals with ASC with and without LD can reflect the differential impact of genetic and environmental risk factors influencing language lateralization and development leading to differential language profiles. However, the question arises whether there are also experience‐dependent influences on cortical asymmetry. Individuals with ASC and LD might show atypical development of lateralization as a consequence of early underuse of specialized language regions. Thus, longitudinal studies are needed to pinpoint the onset and trajectory of atypical development in subgroups on the autism spectrum.

### Origins of Atypical Lateralization

The onset of asymmetric gene expression in the perisylvian region begins in utero [Sun et al., 2005], confirming the involvement of non‐random biological processes in the establishment of asymmetry. Some autism‐risk genes are associated with language impairment [FOXP2: MacDermot et al., 2005; CNTNAP2: Vernes et al., 2008] and brain asymmetry [LRRTM1: Francks et al., 2007]. Nevertheless, the genetic influence on the left hemisphere is limited compared with the prenatal environment, which influences development of the left hemisphere greater than twice that of the right hemisphere [Geschwind et al., 2002]. This is probably due to the left hemisphere's extended period of maturation [Chiron et al., 1997]) which makes it more vulnerable to prenatal environmental perturbations. Recent studies from our group demonstrated that prenatal testosterone exposure is associated with rightward asymmetry of the isthmus of the corpus callosum (a region projecting to cortical language areas) [Chura et al., 2010] and the volume of the PT [Lombardo et al., 2012], which may help explain the known sex differences in rates of language‐related difficulties. ASC in males is associated with the exposure of elevated levels of foetal steroid hormones [Baron‐Cohen et al., 2015] lending support to the theory that testosterone shifts left‐hemisphere functions to the right side [Geschwind and Galaburda, 1985]. However, it remains unclear how prenatal steroid hormones are associated with the atypical pattern of rightward asymmetry in autism. Other prenatal risk factors associated with autism such as maternal infection or gestational diabetes [Gardener et al., 2011] might interfere with left‐hemisphere maturation. These mechanisms are not mutually exclusive; gestational diabetes for example leads to increased foetal testosterone [Morisset et al., 2013]. The underlying mechanisms through which genetic programming and environmental influences interact to give rise to atypical asymmetry in ASC is complex and yet to be established.

### Future Directions

The study of cerebral asymmetry in autism could be extended to subgroups beyond high‐functioning males: it would be important to examine whether individuals with language impairments show more pronounced atypical asymmetries in the same or different areas. Additionally, since neurotypical females are usually less strongly lateralized in language functions [Kansaku et al., 2000; Shaywitz et al., 1995], and given evident differences in brain morphology [Lai et al., 2013b] and cognitive profiles [Lai et al., 2012b] between males and females with ASC, another key direction is to investigate how females with ASC differ in cerebral asymmetry.

## CONCLUSIONS

Both atypically reduced (leftward) and reversed (rightward) asymmetries are present in right‐handed male adults with ASC in persylvian regions. This atypical cortical volumetric asymmetry is associated with early social deficits in childhood. How different patterns of cerebral asymmetry reflect common and subgroup‐specific developmental trajectories in individuals with ASC remains to be clarified. Further research is needed into females with ASC, left‐handed individuals, and different age groups, to establish the timing and mechanisms of the divergence in the establishment of cerebral asymmetry. Different degrees of reductions in leftward lateralization of perisylvian regions potentially constitute a biological underpinning of language delay in ASC, and represent a candidate neurophenotype of ASC. The yet unsuccessful quest for biomarkers for ASC based on recent large‐scale volumetric studies [Haar et al., 2014; Lefebvre et al., 2015] emphasizes the necessity for alternative ways of characterizing brain structure in ASC, and cerebral asymmetries constitute one promising candidate.

## Supporting information

Supporting Information
