# Supplementary material for: Atypically rightward cerebral asymmetry in male adults with autism stratifies individuals with and without language delay
Source: Hum Brain Mapp. 2015 Oct 23;37(1):230–53. doi: 10.1002/hbm.23023 (PMC4913747; doi:10.1002/hbm.23023)
Supplement: Supplementary file 1 — Supporting Information [file HBM-37-230-s001.docx]

**Supplementary Material**

**A) Comparison of Methods**

**Methods**

**Method 1 (Segment then reflect; used in the main analysis as described in the ‘Image segmentation then reflection’ section)**

All original images (N=136) were segmented into grey matter (GM), white matter (WM) and cerebrospinal fluid (CSF) using the VBM8 toolbox. GM segmentations were then rigid-body registered to the MNI template and reflected across the cerebral midline (x=0) using a custom-built script as described in the Materials and Methods section of the main text.

**Method 2 (Reflection then image segmentation)**

We first created a set of mirror images of all simulated T1-weighted images by reflection across the midline of the field of view using the same script as in Method1, thus avoiding further interpolation. For all images (N=136) and their reflected versions (N=136), GM, WM and CSF segmentations were estimated and rigid-body-registered to the MNI template using the VBM8 toolbox. All following preprocessing steps correspond to those described in the Materials and Methods section of the main text.

**Quality Control**

Optimized LI images should theoretically have the same values at homologous voxels in the right and left sides only differing by their sign. However, as the DARTEL preprocessing steps included inevitably interpolation, we did not expect perfect correspondence (i.e., the generation of symmetrical MNI space individual images to estimate LI at each voxel that are perfectly accurate). Comparing the LI images generated from the two methods by the following two indices, as well as one additional post-statistical comparison check, helped select the optimized pipeline for asymmetry-related analyses.

1. Intraclass correlations:

LI images were reflected across the midline (x=0) then multiplied with -1. Intraclass correlations (ICC) were computed between the reflected and non-reflected LI images. Perfect correspondence results in an ICC of 1.

1. Hemispheric LI-difference images:

LI images were reflected across the midline (x=0) then multiplied with -1. The reflected LI images were then subtracted from the non-reflected LI images. Perfect correspondence would result in an image with values of 0 at each voxel.

1. Post-statistical comparisons based on the distributions of t-maps:

Resulting t-maps for the comparisons of the left vs. the right hemisphere of the LI images resulting from method 1 and method 2 were analysed in terms of their distributions. We fitted a Gaussian function ($f\left( x \right)=ae^{-(\frac{{(x-b)}^{2}}{c^{2}})}$) to each distribution and the 95% confidence intervals (CI) of the three fitted parameters were compared pairwise (left vs. right) for each method. Perfect correspondence would result in completely overlapping CIs.

**Results**

1. Intraclass correlations:

Intraclass correlations were calculated between reflected and non-reflected smoothed laterality images for both methods separately. ICCs for single individuals ranged between *r*=0.99–1 for method 1 (segment then reflect) whereas between *r*=0.053–0.96 for method 2 (reflect then segment). Also, the Pearson’s correlation between total GM LIs derived from the left and right side of the LI image was for the sum across all individuals *r*=1.000 for method 1 and *r*=0.709 for method 2.

1. Hemispheric LI-difference images:

Values in LI-difference images for method 1 (*Mean*=0.517, *SD*=15.662) were not significantly different from 0 (*t*=0.385, *p*=0.701). Values in LI-difference images for method 2 (*Mean*=-181.907, *SD*=1243.652) trended towards being significantly different from 0 (*t*=-1.706, *p*=0.09).

1. Post-statistical comparisons based on the distributions of t-maps:

For method 1 there were inclusive overlaps for the three parameters (CI of *a_lef_*_t_ = 218.7 – 231.2 vs. CI of *a_right_* = 221.1 – 231.4; CI of *b_left_* = 0.070 – 1.137 vs. CI of *b_right_* = 0.0872 – 0.1421; CI of *c_left_* = 1.421 – 1.515 vs. CI of *c_right_* = 1.432 – 1.51) (see Figure S1a).


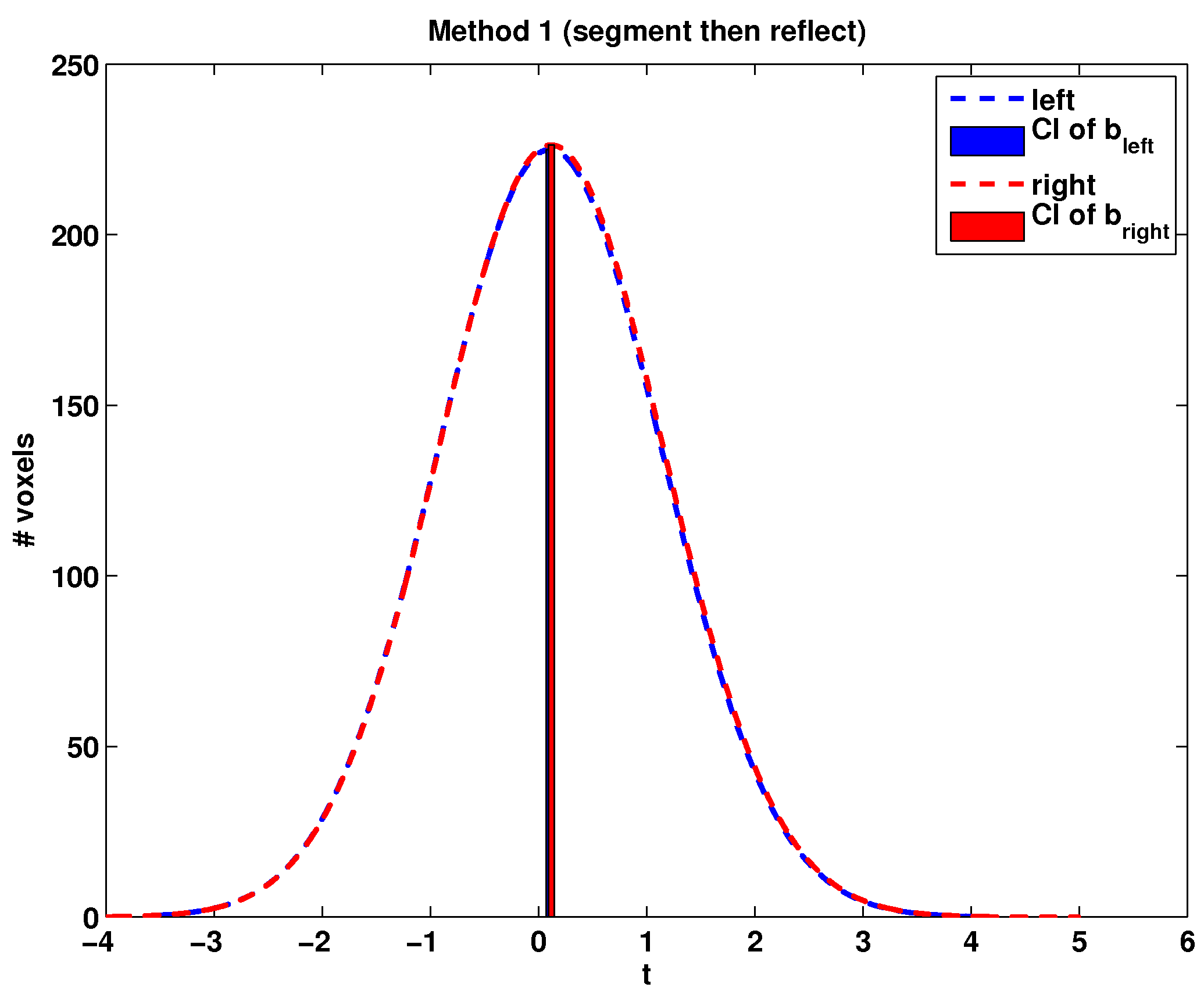


For method 2 there were significantly different CIs for b (CI of *a_left_* = 220.2 – 229.6 vs. CI of *a_right_* = 217.83 – 228.9; CI of *b_left_* = 0.3502 – 0.4002 vs. CI of *b_right_* = -0.0172 – -0.0419; CI of *c_left_* =1.426 – 1.497 vs. CI of *c_right_* = 1.418 – 1.502) (see Figure S1b).


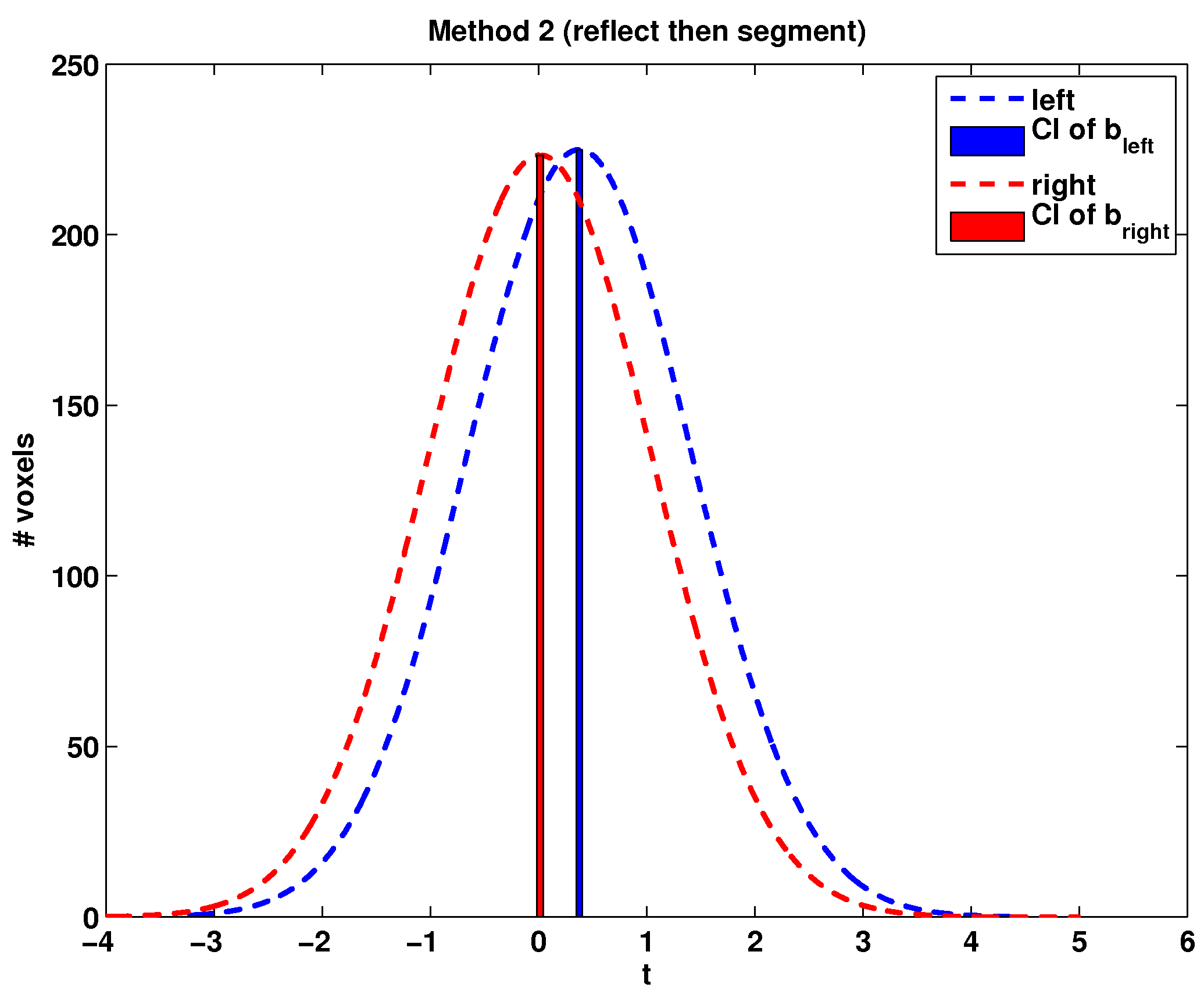


Based on these measures of quality, method 1 (segment then reflect) resulted in more robust data and was deemed to be more optimized to be used in asymmetry-related analyses.

**B) Age- and IQ-matched Sub-Sample**

**Introduction**

An age- and IQ-matched adult subsample was created and preprocessing and analyses were repeated for the functional auditory ROI to investigate whether results remained unaffected.

**Participant characteristics**

Participants comprised 46 controls, 26 individuals with ASC with LD and 41 individuals with ASC without LD. Individuals with ASC and LD and controls did not significantly differ in age (*t*(70)=-1.579, *p*=0.119), VIQ (*t*(70)=-0.88, *p*=0.930) and FIQ (*t*(70)=-1.626, *p*=0.109). Differences remained in PIQ (*t*(70)=-2.933, *p*=0.005).

**Functional Auditory ROI**

Between-group voxel-wise analysis of LI in the functional auditory ROI revealed one significant cluster (cluster size *ke*=354 voxels, cluster-level FDR-corrected *q*=0.026, peak-voxel MNI coordinate [37, -30, 10], *T*=3.54). There was no cluster showing a significant group-by-age interaction.
